# Developing an interpretable model for BCF prediction based on physicochemical descriptors

**DOI:** 10.1093/bib/bbaf631.048

**Published:** 2025-12-12

**Authors:** Taishi Yoshimura, Ayumu Muta, Riku Maeda, Kei Nakayama, Rumi Tanoue, Midori Iida

**Affiliations:** Department of Physics and Information Technology, Kyushu Institute of Technology, 680-4 Kawazu, Iizuka 820-8502, Fukuoka, Japan; Department of Physics and Information Technology, Kyushu Institute of Technology, 680-4 Kawazu, Iizuka 820-8502, Fukuoka, Japan; Department of Physics and Information Technology, Kyushu Institute of Technology, 680-4 Kawazu, Iizuka 820-8502, Fukuoka, Japan; Center for Marine Environmental Studies, Ehime University, 2-5 Bunkyo-cho, Matsuyama, Ehime 790 8577, Japan; Center for Marine Environmental Studies, Ehime University, 2-5 Bunkyo-cho, Matsuyama, Ehime 790 8577, Japan; Department of Physics and Information Technology, Kyushu Institute of Technology, 680-4 Kawazu, Iizuka 820-8502, Fukuoka, Japan

## Abstract

**Aim:**

The bioconcentration factor (BCF) is a key parameter for evaluating the bioaccumulation potential of chemicals. However, experimental measurement of BCF is time-consuming, costly, and ethically challenging due to animal testing. Many existing computational models prioritize predictive accuracy over interpretability. Yet, clarifying the molecular features that govern bioaccumulation is increasingly important.^1^ This study aimed to develop an interpretable regression model for BCF prediction based on physicochemical descriptors.

**Methods:**

Molecular descriptors were computed from SMILES. Several machine learning algorithms were benchmarked, including random forest, and XGBoost. SHapley Additive exPlanations (SHAP) values were used for feature selection and model interpretation, identifying key physicochemical and structural contributors to BCF.

**Results:**

The optimized Gradient Boosting Decision Trees (GBDT) model achieved an R^2^ of 0.57 with 10 selected features and outperformed random forest and linear models. SHAP analysis revealed that Topological Polar Surface Area (TPSA), hydrophobicity (log P), and molecular volume were the most influential descriptors. Higher log P and molecular volume increased BCF, whereas greater TPSA was associated with lower bioaccumulation.

**Conclusions:**

This study highlights an interpretable machine learning framework for predicting BCF based on physicochemical descriptors. By linking molecular features with bioaccumulation potential, the model supports transparent and reliable in silico assessment, contributing to ethical and cost-effective chemical risk evaluation.

**References:**

1. OECD, ‘(Q)SAR Assessment Framework: Guidance for the regulatory assessment of (Quantitative) Structure Activity Relationship models and predictions - Second edition, OECD Series on Testing and Assessment No. 405,’ OECD Publishing 2024.

